# Management of elderly patients with esophageal squamous cell cancer

**DOI:** 10.1093/jjco/hyac067

**Published:** 2022-04-30

**Authors:** Yasuo Hamamoto, Kentaro Murakami, Ken Kato, Yuko Kitagawa

**Affiliations:** Keio Cancer Center, Keio University Hospital, School of Medicine, Tokyo, Japan; Department of Frontier Surgery, Graduate School of Medicine, Chiba University, Chiba, Japan; Department of Head and Neck, Esophageal Medical Oncology, National Cancer Center Hospital, Tokyo, Japan; Department of Surgery, Keio University School of Medicine, Tokyo, Japan

**Keywords:** geriatric oncology, radiotherapy, chemoradiotherapy, esophagectomy, esophageal cancer

## Abstract

This review focuses on the treatment about elderly esophageal cancer to clarify the current situation regarding our clinical question. Although there are several reviews about elderly esophageal cancer treatment, there are fundamental differences between Japan and the rest of the world. Two main differences are raised: histological differences and treatment strategies for resectable patients. We overview each status according to following clinical questions. First, there are no established evaluation criteria for frail. Second, selection criteria for surgery or non-surgery are not established. Third, few specific treatments for elderly patients (EPs) are investigated. In conclusion, there are many reports about treatment of esophageal squamous cell carcinoma for EPs, although treatment strategy is still controversial. We have to consider well-designed prospective trial to confirm specific treatment strategy according to each stage.

## INTRODUCTION

Esophageal cancer is the sixth leading cause of cancer death globally and the ninth leading cause of cancer death in Japan (1). This cancer is associated with extensive treatment requirements and poor prognoses. Endoscopic procedures have increasingly been used in the treatment of premalignant and early tumors. Curative treatment consists of neoadjuvant chemotherapy (NAC) or neoadjuvant chemoradiotherapy (NACRT) followed by extensive surgery for locally advanced disease (2–4). For metastatic cases, systemic chemotherapy is available for palliative intent. Esophageal squamous cell carcinoma (ESCC) is the most common histological subtype of esophageal cancer, particularly in Japan. Endoscopic approach for tiny disease is attractive approach as less invasive approach. It is of importance as diagnostic value and minimum invasive option. Standard curative treatment in Japan consists of NAC followed by surgery (NAC-S) for patients who can tolerate radical treatment (5,6). There are many treatment options for elderly esophageal cancer patients; however, the main curative treatments are too radical for vulnerable and frail patients (7–9). Modified NAC-S, surgery alone and definitive CRT (DCRT) followed by selected surgery are major options for fit or vulnerable patients. Radiotherapy (RT) alone has also been established for the treatment of elderly patients (EP). Systhemic chemotherapy for metastatic disease as palliative intent also need to cosider some modification according to the risk benefit balance of symptom relief and toxicities. To select optimal treatments, multi-disciplinary teams use geriatric assessments to provide objective information; however, standard tools for ESCC are not available in Japan (10,11).

Many studies have tried to resolve the above clinical questions. Unfortunately, there are no definite answers (12). Several investigators have published review articles about treatment strategies for EP. These reviews are helpful when considering treatment strategies for the elderly. Although these articles scientifically describe and clarify the current status of treatments, there are fundamental differences between Japan and the rest of the world (9,13,14). There are two main differences: histological differences and treatment strategies for resectable patients. In Japan and China, most patients have squamous cell cancer, whereas in Western countries, most patients have adenocarcinoma. More importantly, the Japanese standard treatment for resectable ESCC is NAC-S; however, other countries use NACRT followed by surgery as the standard of care. Several clinical questions are raised in practice. First, there are no established selection criteria (Fit/Vulnerable/Frail) in each stage according to treatment. Second, surgery or non-surgery is still controversial even in non-EPs. Third, few specific treatments are investigated for EPs in ESCC. This review focuses on the Japanese standard of care to clarify the current global situation regarding our clinical question.

## Stage 0

Standard treatment for stage 0 disease is endoscopic resection followed by additional treatment, if non-curative disease. Less invasive option is no additional treatment for vulnerable patient. Several reports suggested Charlson-Comorbidity Index (CCI) as reliable assessment tool for selection. Although additional treatment after incomplete treatment (most of them are stage 1 disease) is routinely recommended, which should not be mandatory for EP. Both surgery and non-surgical treatments are candidate when considering additional curative options if the patient’s natural life expectancy is enough long. The natural prognosis of each patient by precise assessment is important when considering whether additional curative treatments are recommended.

Recently, endoscopic submucosal dissection (ESD) has become an accepted treatment for early esophageal cancer. It is a less invasive curative option, especially for EP. The indication of ESD is considered – the lesions invading up to the muscularis mucosae or only slightly infiltrating the submucosa up to 200 μm (MM/SM1), as well as lesions limited to the mucosal epithelium or the lamina propria mucosa (EP/LPM). ESD was generally applied for lesions more than 15 mm in size, particularly over 20 mm. When ESD results were diagnosed with the depth of the lesion to be submucosa (SM), lymphovascular invasion or positive findings of vertical margin, additional treatment such as surgery or CRT was considered. The safety and efficacy of ESD for ESCC were reported by several investigators. Short-term outcomes indicated that it was safe and effective although EP had more complications as well as a significantly higher rate of usage of anti-platelet agents or anticoagulants (15–18). Two investigators also reported the long-term survival was lower in EP. Noh et al. (19) reported no patients over 75 years were selected for additional surgery; however, Ishido et al. (20) reported several treatments including surgery. They suggested the CCI ≥ 2 was an independent prognostic factor for survival in the elderly group. Miyamaoto et al. (21) reported no patient was selected for additional treatment including CRT. The differences between previous reports were the cut-off age of EPs. Miyamoto et al. defined endoscopically EP as those over 80 years old. Different from previous reports, they reported the 3-year overall survival (OS) and disease-specific survival were favorable in both groups. Nakajo et al. (22) reported unique and clinically important data in a multicenter retrospective study. They clarified who required additional treatments after non-curative ESD and post-ESD survival in EP. They also reported that follow-up strategy without additional treatment after non-curative ESD may be acceptable in high-risk EPs, especially for those with CCI ≥ 2. This information clarified the unmet clinical need to avoid overtreatment in EP.

In conclusion, endoscopic resection is minimum invasive treatment with diagnostic value, although there are some incomplete results. For vulnerable patients, treatment completion including additional invasion is not necessary. This strategy for non-curative resection is also applicable for both ESD and classical endoscopic mucosal resection.

## Stage I

Standard treatment for stage I disease is both surgery and CRT. Selection criteria for surgery or non-surgery are not established. Less invasive option is RT alone or modified CRT. Standard treatment or optional treatment is selected by chronological age, physical condition and patient preference. Recently, two important studies were reported from the Japan Clinical Oncology Group (JCOG). JCOG0508 conducted by Minashi et al. (23) investigated prior endoscopic treatment followed by selective CRT. This strategy is quite unique to avoid overtreatment using endoscopic treatment as a diagnostic value. This strategy seems ideal to avoid over-treatment, especially for EP. However, there are several unresolved problems in this radical endoscopic approach. Most important problem is treatment delay due to mechanical ulceration. Subsequent CRT after endoscopic treatment is risk of esophageal stenosis. We need to wait until ulceration improve. The JCOG0502 study conducted by Kato et al. (24) compared surgery and CRT with stage I ESCC. This study concluded that CRT was non-inferior to surgery and should be considered for the treatment of stage I ESCC. Non-surgical treatment options seem preferable, especially for EP. Although this study consists of non-EPs, this information should be shared with esophageal cancer specialists and patients and families to discuss what kind of treatments are favorable for each patient. Both JCOG0508 and JCOG0502 approaches are recommended for endoscopically resectable cases, however, there are several controversy to judge which approach is appropriate. RT in patients over 80 years old is known as safe and effective treatment. Actual 2- and 5-year survival rates of RT is 24% and 5%. (49).

## Stage II/III (non-T4)

Standard treatment for stage II/III (non-T4) is NAC followed by surgery. Definitive CRT is attractive organ preservation option to avoid planned surgery. However, residual disease after CRT and local recurrence is need to consider salvage surgery. Salvage surgery is much invasive than planned surgery especially for EP. Less invasive option is esophagectomy without NAC if feasible. RT alone or modified CRT is also alternative option for vulnerable patients. Unfortunately, there are no established criteria to select treatment strategy for these treatments.

### Stage II/III (non-T4): surgery

Esophagectomy remains the definitive curative treatment of ESCC and prompt symptom relief from dysphagia, although it is an invasive procedure with cardiac and pulmonary complications being a major cause of early mortality and morbidity. The impact of advanced age on esophagectomy outcomes is controversial, although it is safe and effective in selected patients. Recent advances in surgery have changed factors so that age alone is not an exclusive factor for surgery candidates (25–39). Many investigators have emphasized that the physiological condition of the patient is important; however, appropriate selection criteria have still not been established.

Three systematic reviews were conducted including an analysis assessing esophagectomy for EP. Markar et al. (40) reported EPs were at increased risk compared with non-EPs with pulmonary (21.77% vs. 19.49%; pooled odds ratio 1.49; 95% confidential interval (C.I.) = 1.29–1.71; *P* < 0.05) or cardiac complications (18.7% vs. 13.17%; pooled odds ratio = 2.06; 95% C.I. = 1.75–2.41; *P* < 0.05), and perioperative mortality (7.83% vs. 4.21%; pooled odds ratio 1.87; 95% C.I. = 1.54–2.26; *P* < 0.05) following esophagectomy. They showed reduced cancer-related 5-year survival compared with non-EP (34.4% vs. 41.8%; pooled odds ratio 0.75; 95% C.I. = 0.64–0.89; *P* < 0.05). There were no significant differences between the groups regarding the length of hospital stay, incidence of anastomotic leak or conduit ischemia. Han et al. (41) conducted a meta-analysis to compare the clinical outcomes of esophagectomy between EP and non-EP. They also found that esophagectomy for EP had a higher risk of in-hospital mortality (odds ratio 2.00, 95% C.I. = 1.28–3.13; *P* = 0.002), higher incidence rates of cardiac (OR 1.55, 95% C.I. = 1.10–2.20; *P* = 0.01), or pulmonary complications (OR 1.57, 95% C.I. = 1.11–2.22; *P* = 0.01), and lower survival rates (odds ratio hazard ratio 2.66, 95% C.I. = 1.65–4.28; *P* < 0.001). However, there was no significant difference in the overall postoperative complication rate (OR 1.40, 95% C.I. = 0.82–2.40; *P* = 0.22) and the incidence of anastomotic leak (OR 0.92, 95% C.I. = 0.58–1.47; *P* = 0.73). Recently, Mantziari et al. conducted a more precise analysis. They also showed no significant difference in anastomotic leakage rates as well as a higher incidence of pulmonary (median incidence: EP 20% vs. non-EP 16%) and cardiovascular complications (median incidence: EP 15.6% vs. non-EP 7.0%). Postoperative mortality was significantly higher for EP (7.9% for EP vs. 3.4% for non-EP) (42). Although a systematic review reported a trend for high mortality for EP with surgery, a recent study from Japan showed outstanding results (43). Miyata et al. (33) reported comparable mortality, even in EP, although they had a higher frequency of postoperative pulmonary and cardiovascular complications. They concluded the lower overall survival rate in EP compared with non-EP was due to a less aggressive treatment. Kanda reported a propensity score-matched analysis. They revealed that long-term survival was comparable between EP and non-EP (35). Motoyama et al. compared esophagectomy and CRT using the National Database of hospital-based cancer registries in Japan. They revealed that esophagectomy for clinical stage II/III patients was significantly associated with better survival (adjusted hazard ratio: 0.731) (95% C.I. = 0.645–0.829, *P* < 0.001) in the ≥75 years group but not in the ≥80 group, when compared with CRT. These data suggest that selected EPs aged 75–80 years require precisely selected treatment strategies (36), however, those Japanese reports lack selection criteria. Most important clinical question is still unresolved.

Recently, two important studies were reported as new standard of care in non-EPs. One of the study is additional nivolumab after NACRT followed by surgery (118). They concluded additional nivolumab shows superior efficacy over placebo in disease-free survival. Subgroup analysis of ages is not affected efficacy. Although this study did not include EP, additional immune-check point inhibitor is of importance to evaluate for EP. Another study is new NAC triplet regimen from JCOG (119). JCOG 1109 shows triplet regimen is superior efficacy compared to doublet regimen. This non-radiation strategy is attractive to improve treatment results, however, it seems too toxic for EP. This new strategy is also need to consider for EP.

The right- and left-approach open esophagectomies remain the general procedures for patients with operable thoracic esophageal carcinoma. The choice between the two methods is controversial. Liu et al. (43) investigated a retrospective comparison with propensity score matching analysis. Both the OS and DFS were significantly better in the right-approach group, along with better lymph node resection and a lower incidence of recurrence. However, increased incidences of postoperative pneumonia, respiratory failure and sub-clinical anastomotic leak were found in the right-approach group, although the perioperative mortality was similar between groups. Chen et al. (44) also compared the perioperative outcomes and long-term survival rates of the McKeown and Sweet procedures in patients with esophageal cancer. Propensity score matching was used to balance the clinical characteristics of patients who underwent the different surgical approaches. Patients younger than 70 years who underwent the McKeown procedure had a better OS than those in the Sweet group. However, no significant differences in DFS and OS were observed between two approaches for EPs.

Sugita reported that minimally invasive esophagectomy for EPs was a safe and feasible modality (45).

### Stage II/III (non-T4): non-surgical treatments

RT has long been considered as the first-choice treatment for patients over 80 years old (46). Several investigators reported RT is a safe and effective treatment in patients over 80 years old (47). A pattern of care study (PCS) suggested age was not a significant prognostic or risk factor for patients in the non-surgery group treated with RT (48). Kawashima conducted a prospective trial of RT for patients aged 80 years or older with ESCC (49). The primary endpoint was CR rate, which was achieved by 61% (31/51). The median survival time was 30 months and the 3-year OS rate was 39% (95% CI = 24–52%). Their study indicated that RT in octogenarians was comparable to those in non-EP, although there were no control data. Huan et al. (50) reported a retrospective population-based study using The Surveillance, Epidemiology and End Results (SEER) database. The improvement of RT in esophageal cancer was only significant in localized and regional stage patients.

A recent PCS revealed CRT is common for ESCC, but those patients over 75 years were more likely to be treated by RT only (51). DCRT is the preferred curative non-surgical treatment modality. Several studies reported that age alone was not a decisive factor, but that careful attention should be paid to patient selection, however, there are no definite selection criteria currently (28,52–59).

There were also negative reports of CRT. Takeuchi reported a retrospective analysis showing significant inferior efficacy, even in selected patients (60). Chen also reported CRT may not improve the survival of elderly ESCC patients (61). Jingu suggested CRT should be carefully selected (62). Lu et al. (63) recommended customized treatment based on the CCI. Some modifications of CRT warrant investigation. Ji et al. (64) reported an RCT from China investigating RT alone vs. RT with S-1. The CRT group had a significantly higher complete response rate than the RT group (41.6% vs. 26.8%; *P* = 0.007) as well as a significantly higher 2-year OS rate (53.2% vs. 35.8%; HR, 0.63; 95% C.I. = 0.47–0.85; *P* = 0.002). There were no significant differences in the incidences of grade 3 or higher toxic effects between the two groups (9.5% vs. 2.7%; *P* = 0.01). A limitation of this trial was how to select candidate patients in the target population and patients with renal dysfunction with intolerance to S-1. Kawamoto et al. (65) reported the clinical impact of CRT with docetaxel for renal dysfunction. These optional treatments for patients with poor renal function are important for EPs.

## Stage II/III (clinical T4)

Standard treatment for locally advanced T4 disease is definitive CRT. Another option for vulnerable patients is modified CRT or RT alone. In T4 cases, most of the patients suffer severe symptom by tumor invasion to adjacent organs. Symptom relief by intensive treatment is of importance. Definitive CRT is attractive for its anti-tumor effect; however, curability is generally low. On the other hand, toxicity of CRT need to consider especially for EP. We often experience treatment-related death by too intensive treatment. Modified CRT or RT is generally selected for EP; however, efficacy is relatively low and symptom relief is also modest. Patients in this category are generally poor outcome with several complications. Assessment of natural life expectancy and symptom relief is important. Unfortunately, there are little reports about T4 disease for EP.

Previous reports for EP mainly consist of resectable disease. It is important to consider supportive care for these patients category.

## Stage IVb

Very few studies have investigated about metastatic disease in EP with ESCC. Tang et al. (66) constructed a model to predict the cancer-specific survival of metastatic esophageal cancer patients. They constructed a nomogram and a corresponding risk classification system, which had the potential to assist in patient counseling and decision making. Recently, additional immune check point inhibitors improved overall survival in non-elderly patients (120). The data suggested manageable safety profile for eligible patients. These non-cytotoxic treatments could be of importance for EP. Further evaluation is warranted.

## Patient selection and evaluation

There are no established way to select fit healthy, vulnerable or frail. Assessment by geriatric assessment is one of available candidate for selection method. Assessment of body composition is also widely reported evaluation especially ESCC. Although geriatric assessment has a role in guiding treatment decisions in early and advanced settings, few studies have conducted geriatric assessments for ESCC. Sarcopenia is a poor prognostic factor in patients with ESCC. EPs have the potential risk for sarcopenia status. There has recently been increased interest in the assessment of body composition in patients with esophageal cancer for the purpose of nutritional evaluation and prognostication. Geriatric assessments and sarcopenia are good candidate for selection criteria for treatment, however, they need to be validated prospectively.

### Geriatric assessment

Yamamoto et al. (67) investigated the risks of postoperative complications before surgery and found an association between postoperative delirium and clinicopathological factors, including comprehensive geriatric assessment (CGA). They found postoperative delirium was significantly associated with a low mini-mental state examination (MMSE) score and high Geriatric Depression Scale 15 (GDS15) score, which are components of the CGA and psychiatric disorders. Hirpara et al. (68) used multiple physiotherapy tests (6-min walk, gait speed, hand-grip strength), risk stratification (CCI, Revised Cardiac Risk Index, Modified Frailty Index) and quality of life questionnaires for risk assessment prior to thoracic surgery. Regarding the risk stratification questionnaires, the Modified Frailty Index may be useful at predicting outcomes as per this feasibility study. Shimada et al. (69) surveyed a questionnaire comprising the EORTC QLQ-C30 and G8 in those undergoing preoperative chemotherapy before esophageal cancer surgery. They found the QOL scores were decreased in terms of physical functions but improved in terms of esophageal cancer symptoms and mental functions. These results suggest that the alleviation of symptoms contributed to the improvements in mental health.

### Assessment of body composition and sarcopenia

Many reports showed the impact of sarcopenia on survival (70–79). Those analyses underwent before or after neoadjuvant treatment (NAT) followed by surgery are increasing (80–85). Boshier et al. (86) conducted a systematic review and meta-analysis of sarcopenia for ESCC who received surgery. Twenty-nine studies reported a clinically useful assessment tool that could support decision-making in patients with esophageal cancer. Therefore, the assessment of body composition has the potential to become a clinically useful tool that could support decision-making in patients with esophageal cancer. The current evidence is however weakened by inconsistencies in the methods of assessing and reporting body composition in this patient group. Deng et al. (87) also conducted a comprehensive systematic review and meta-analysis of 11 studies. They found preoperative sarcopenia was an independent unfavorable prognostic factor for ESCC. Jin et al. (88) conducted a systematic review with meta-analysis in patients receiving neoadjuvant therapy for esophageal cancer. They conducted a subgroup analysis of overall survival based on sarcopenia before NAT and after NAT. Interestingly, there was no statistically significant difference in OS in the before NAT group, but the other group showed the opposite. The patients diagnosed with sarcopenia following NAT had a poor prognosis. However, a very recent systematic review revealed the impact of preoperative sarcopenia including NAT. Sarcopenia before NAT was not statistically different but sarcopenia following NAT had a poor prognosis. Ida and Nishigori reported sarcopenia may be a predictor of pulmonary complications after esophagectomy (89,90).

There are several interventions that can improve nutrition status. Oguma et al. (91) used nutritional support and rehabilitation in ESCC patients and suggested these strategies might help to prevent postoperative pulmonary complications and improve long-term outcomes. Ishikawa and Wu also revealed that nutrition counteracted sarcopenia development during treatment (92,93). Takesue et al. (94) reported a prospective RCT of enteral nutrition after esophagectomy and revealed the nutrition group suppressed weight loss and had a reduced incidence of pneumonia. Yamamoto et al. (95) also suggested that the administration of ghrelin after esophagectomy increased oral food intake and attenuated weight loss together with the maintenance of lean bodyweight.

The impact of DCRT on sarcopenia patients is controversial. Sato, Xu and Takahashi reported poor nutrition status was a significant negative prognostic factor of survival (96–98). However, Ma et al. (99) reported sarcopenia before CRT did not affect survival. They reported that the post-CRT sarcopenia group had a shorter survival. Another nutrition index was reported by several investigators. Yamana, Kubo and Kouzu reported the geriatric nutrition risk index (GNRI) was a useful marker that could be used to assess the nutritional status and predict the development of postoperative respiratory complications (100–102). Takahashi reported preoperative osteopenia was associated with poor survival and recurrence in patients undergoing esophagectomy (98).

**Table 1:** Basic treatment strategy according to stage and elderly status

	Fit healthy	Vulnerable	Frail
Stage 0	Endoscopic treatment +/− additional treatment (additional treatment if non-curative disease)	Endoscopic treatment (additional treatment is not mandatory)	Observation
Stage I	1) Esophagectomy 2) CRT 3) Endoscopic treatment +/− additional treatment	1) RT alone 2) Modified CRT 3) Esophagectomy if feasible	BSC
Stage II/III (non-T4)	1) Neoadjuvant chemotherapy + esophagectomy 2) CRT +/− esophagectomy 3) CRT + esophagectomy + nivolumab	1) RT alone 2) Modified CRT 3) Esophagectomy if feasible	1) BSC 2) Stent
Stage II/III (cT4)	CRT	1) RT alone 2) Modified CRT	1) BSC 2) Stent
Stage IVb	1) Chemotherapy 2) BSC	1) BSC 2) Modified chemotherapy	BSC

BSC: Best supportive care, CRT: Chemoradiation, RT: Radiation.

CRT: chemoradiation, ESCC: esophageal squamous cell carcinoma, NAC: neoadjuvant chemotherapy, RT: radiation.

## New optimal treatments expected for elderly patients

Several new technologies are considered as preferred options for EPs. Proton beam therapy can irradiate a targeted tumor, whilst sparing normal tissue irradiation compared with X-ray therapy. A retrospective single institutional study by Ono et al. (103) showed promising results. They reported 54 patients including 70% inoperable cases with a 5-year survival rate of 56.2%. Near-infrared photoimmunotherapy (NIR-PIT) is a newly developed cancer treatment that uses a highly targeted monoclonal antibody conjugated to a photo absorber (104). A first-in-human phase 1/2 clinical trial of NIR-PIT using cetuximab-IR700 (RM-1929) targeting epidermal growth factor receptor (EGFR) in patients with inoperable head and neck squamous cell cancer has been successfully concluded. A global phase 3 clinical trial has begun for head and neck cancer. NIR light can be administered endoscopically, and early phase trial targeting esophageal squamous cell cancer has also begun. Therefore, NIR-PIT might be a promising candidate for treating ESCC, especially EP. OBP-301 (Telomelysin) is an attenuated type-5 adenovirus that contains the human telomerase reverse transcriptase promoter that regulates viral replication (105). OBP-301 sensitizes human cancer cells to ionizing radiation by inhibiting DNA repair, whereas in contrast, radiation enhances coxsackievirus and adenovirus receptor-mediated OBP-301 infection. A phase I dose-escalation study of OBP-301 with RT was conducted in 13 histologically confirmed esophageal cancer patients. No dose-limiting toxicities were observed in the study and OBP-301 was safely combined with RT at the highest dose. Only the frequency of fever was higher compared with the OBP-301 monotherapy trial. Of 13 patients, eight patients had local complete responses. The clinical CR rate was 83.3% in stage I and 60.0% in stage II/III. Multiple courses of endoscopic intratumoral OBP-301 injection with RT are feasible and provide clinical benefits in patients with esophageal cancer unfit for standard treatments. EGFR is overexpressed in 30–70% of patients with ESCC and is associated with a poor prognosis and inferior responses to conventional treatment. Icotinib, an oral EGFR inhibitor, was reported to markedly inhibit the proliferation of a human epidermoid squamous cancer cell line expressing a high level of EGFR. A randomized phase 2 trial was conducted and icotinib and RT were well tolerated and showed survival benefit (106). A phase III trial to further identify the efficacy of icotinib with RT is forthcoming.

## Discussion

ESCC represents a significant disease burden in the older population. It is a highly morbid condition with the older population being especially vulnerable to the complications of dysphagia, malnutrition and sarcopenia. As with many other cancers, trial data disproportionally represent a younger, better performance status population, although certain historical and increasing numbers of newer trials are assessing this disease specifically in the older population. We summarized the current Japanese treatment strategies for ESCC for EP (Table 1).

In locally advanced operable stage (stage II/III, non-T4 disease), the selection of treatment strategy is relatively complex. Both surgery and definitive CRT are good candidates as curatives; however, no clear selection criteria exist to date. If patients are suitable for NAT, NAC-S or NACRT, the estimation of long-term survival will differ. Recently, active surveillance after CRT has been considered (107). DCRT followed by salvage was also reported as a safe and effective option for locally advanced operable stage (108). Noordman et al. (109) initiated an RCT comparing active surveillance vs. standard esophagectomy after NACRT. Although these studies were conducted in non-EP, avoiding radical treatment strategy is a critical issue for EP.

There is also the important issue of cost. Older patients and those with higher severity of illness are associated with worse outcomes. They tend to have higher perioperative mortality, readmission rates, hospital costs and require more postoperative care. With increasingly scrutinized health care costs, these data provide guidance for more careful patient selection (110). Costs dependent on treatment type have also been reported in Japan. The median cost of surgery was more than double that of RT. Comparing the cost-effectiveness of treatment, surgery has the highest average and wide range of costs including several outliers. The cost of RT was remarkably lower than costs associated with other treatment modalities. CRT and modified CRT had equivalent costs. Surgical expenses tended to be higher in low-volume centers (111).

Malnutrition is the most common complication of esophageal cancer with a poor prognosis. Several attempts to improve physical condition are reported and ongoing including whole-course nutrition management on the CRT. Qiu et al. (112) conducted an RCT to compare an intervention group and control group. They revealed whole-course nutrition management improved the nutritional status of patients with esophageal cancer. These attempts are essential interventions, especially for EP.

How to share this information with caregivers and patients is difficult. Shared decision-making is essential for those who are applicable for several treatment options. Evidence, preferences and recommendations need to find the right balance for patient care (113). There are several other concerns regarding decision making for treatment. Some investigations of human behavior suggested that EPs prefer limited options opposed to multiple treatment choices in the process of decision making (114). Recent advances in the research of patient preferences showed that although there was heterogeneity among patients, they were willing to accept a lower 5-year OS to achieve an improved outcome that avoided esophagectomy (115,116). Geriatric oncology has provided information for oncologists in selecting the optimal treatment including patient preference; however, information for medical education and clinical practice is inadequate in Japan (117).

In conclusion, there are many reports about treatment of ESCC for EP although those data are still controversial. There are several advantages and disadvantages according to each treatments (Table 2). We have to consider well-designed prospective trial to confirm these specific treatment strategy.

**Table 2:** Lists of advantage or disadvantage according to treatment options in ESCC

	Advantage	Disadvantage
Endoscopic resection	Minimum invasive treatment Diagnostic value	Incomplete treatment if disease is deep or lympho-vascular metastasis Treatment delay if additional treatment is CRT
Surgery	Curability is relatively high Prompt symptom relief from dysphagia	Invasive treatment for elderly patients High mortality and morbidity Gastrointestinal disorder due to reconstruction
NAC followed by surgery	Definitive curative treatment Treatment strategy can change after NAC response	Toxicity of chemotherapy Total treatment completion is difficult for elderly patients
CRT	Organ preservation strategy Treatment can terminate if treatment is not feasible	Toxicity of chemotherapy and radiation Some modification is essential Symptom relief needs at least several weeks Treatment completion is difficult for elderly patients Salvage surgery after CRT is difficult
RT	Less invasive treatment Modest effect for symptom relief Treatment completion rate is relatively high	Curability is low for advanced disease Symptom relief needs at least several week Radiation toxicity

## Conflict of interest

Y.H. received funding from Ono Pharmaceutical Co. K.M. has nothing to disclose. K.K. reports research grants from Ono Pharmaceutical, Shionogi, MSD, Merck Serono, Beigene, Oncolys Biopharma, Chugai Pharmaceutical and Bayer outside the submitted work. Y.K. received lecture fees from Asahi Kasei Co, Ltd; Taiho Pharmaceutical Company, Ltd; Chugai Pharmaceutical Company, Ltd; EA Pharma Company, Ltd; Yakult Honsha Company, Ltd; Otsuka Pharmaceutical Company, Ltd; Otsuka Pharmaceutical Factory Inc, Shionogi & Company, Ltd; Astellas Pharma, Inc; Dainippon Sumitomo Pharma Company, Ltd; Taisho Toyama Pharmaceutical Company; Ono Pharmaceutical Company; Nihon Pharmaceutical Co, Ltd; Sanofi K.K., Eisai Company, Ltd and Kaken Pharmaceutical Company, Ltd. Y.K. received Grants for research expenses and scholarship from Taiho Pharmaceutical Company, Ltd; Chugai Pharmaceutical Company, Ltd; Yakult Honsha Company, Ltd; Daiichi Sankyo Company, Ltd; Merck Serono Company, Ltd; Asahi Kasei Company, Ltd; EA Pharma Company, Ltd; Otsuka Pharmaceutical Company, Ltd; Takeda Pharmaceutical Company, Ltd; Otsuka Pharmaceutical Factory, Inc; Shionogi Company, Ltd; Kaken Pharmaceutical Company, Ltd; Kowa Pharmaceutical Company, Ltd; Astellas Pharma, Inc; Medicon, Inc; Dainippon Sumitomo Pharma Company, Ltd; Taisho Toyama Pharmaceutical Company, Ltd; Kyouwa Hakkou Kirin Company, Ltd; Pfizer Japan, Inc; Ono Pharmaceutical Company, Ltd; Nihon Pharmaceutical Company, Ltd; Japan Blood Products Organization Medtronic Japan Company, Ltd; Sanofi K.K., Eisai Company, Ltd; Tsumura & Company, Ltd; KCI Licensing, Inc; Abbott Japan Company, Ltd and Fujifilm, Toyama Chemical Company, Ltd. Y.K. worked as a director of endowed chair supported from Taiho Pharmaceutical Company, Ltd; and Chugai Pharmaceutical Company, Ltd.

## Supplementary Material

To_reviewer_hyac067
