# Supplementary material for: Management of elderly patients with esophageal squamous cell cancer
Source: Jpn J Clin Oncol. 2022 Apr 30;52(8):816–24. doi: 10.1093/jjco/hyac067 (PMC9354502; doi:10.1093/jjco/hyac067)
Supplement: To_reviewer_hyac067 [file to_reviewer_hyac067.doc]

To reviewer

Many thanks for your kind remarks. We appreciate your important comments. We have correct our manuscript as follows. We answer point-to point to your comments.

**Additional comments**

**1. We are sorry for not pointing out in the previous review. The beginning of the introducsion seems focus on stage II/III disease. Introduction should cover the all stages.**

**ANSWER**

**Thank you for your comments. Actually most of investigation about elderly esophageal cancer is locally advanced disease. So we describe mainly stage II/III disease, however we review all stage in this manuscript. We agree to mention more comments about other stage. So, we have add several sentences to mention early disease and metastatic disease.**

**We add following sentences**

**-Endoscopic procedures have increasingly been used in the treatment of premalignant and early tumors. Curative treatment consists of neoadjuvant chemotherapy (NAC) or neoadjuvant chemoradiotherapy (NACRT) followed by extensive surgery for locally advanced disease [2, 3, 4]. For metastatic cases, systemic chemotherapy is available for palliative intent.**

**Endoscopic approach is attractive approach as less invasive approach. It is of importance as diagnostic value and minimum invasive option.**

**Systemic chemotherapy for metastatic disease as palliative intent need to compare the benefit of symptom relief and toxicities.**

**2．The authors describe about ESD for stage 0 disease. However, non-curative resection is not limited to ESD but applicable to endscopic resection including EMR.**

**ANSWER**

**Thank you for your thoughtful suggestion. This comments is really important, however recent reports are limited in ESD. I also think non-curative resection is also applicable in EMR. Unfortunately there are no report about data of elderly patients in EMR. We add following sentences to emphasize the strategy of non-curative resection would be also applicable in EMR case.**

**We add following sentences**

**These strategy for non-curative resection is also applicable both ESD and classical endoscopic mucosal resection.**

**3. For stage I disease, the authors refer to two studies, JCOG0508 and JCOG0502. It is not clear which is recommended for EP if the lesion can be resected endscopically. Otherwise, is this also controversial?**

**ANSWER**

**Thank you for pointing out most critical problems. This clinical question is quite important to discuss. Both study is targeted same stage. For stage I with endoscopically resectable cases, minimum invasive strategy seems attractive, however non-curative prognosis is poor. In these aspect, endoscopic approach for non-curative results are not mandatory recommended. Non curative resection for EP seems acceptable for EP because the non-invasive, less toxic method is of importance for vulnerable patients. It could be said these problems are still controversial and difficult to conclude. We though further investigation is warranted.**

**We have add following sentences to emphasize there remains controversy what is recommended for stage I cases.**

**Both JCOG0508 and JCOG0502 approach is recommended for endoscopically resectable cases, however there are several controversy to judge which approach is appropriate.**

**3. For stage II/III (T4), it should be described more about "What can be ecpected by the mild CRT or RT for EP?", considering the expected cure rate and complication rate regardless age and balance of merit and demerit of mild treatment option for EP.**

**Thank you for your suggestion. Most of T4 disease in elderly patients raise clinical controversy. Definitive CRT is more efficacy but too toxic. Modified CRT is attractive for elderly patients as less toxic option although efficacy is lower than standard CRT. RT alone is reasonable approach for EP, however anti-tumor effect is limited. T4 disease invading aorta, trachea or bronchus is life-threating situation with severe symptom. Curability is generally low (less than 10% for non-elderly patients). On the other hand, morality of definitive CRT is high (10% or more). We have to consider well-balanced assessment and recommendation for these patients. In general, advanced care planning with patients and family what kind of approach is acceptable for each patients. We do not have appropriate reference about T4 treatment in elderly patient, we have add our recommendation about these category of patients as personal considerations.**

**We have add following comments in this manuscript.**

**In T4 cases, most of the patients suffer severe symptom by tumor invasion to adjacent organs. Symptom relief by intensive treatment is of importance. Definitive CRT is attractive for its anti-tumor effect, however curability is generally low. On the other hand, toxicity of CRT need to consider especially for EP. We often experience treatment related death by too intensive treatment. Modified CRT or RT is generally selected for EP, however efficacy is relatively low and symptom relief is also modest.**
